# Finite-gain $\mathcal{L_{\infty}}$ stability from disturbance to output of a class of time delay system

**DOI:** 10.1186/s13660-016-1290-y

**Published:** 2017-01-13

**Authors:** Ping Li, Xinzhi Liu, Wu Zhao

**Affiliations:** 1 College of Computer Science and Technology, Southwest University for Nationalities, Chengdu, 610041 P.R. China; 2 Department of Applied Mathematics, University of Waterloo, Waterloo, Ontario N2L 3G1 Canada; 3 School of Management and Economics, University of Electronic Science and Technology of China, Chengdu, 610054 P.R. China

**Keywords:** finite-gain $\mathcal{L_{\infty}}$ stable from disturbance to output, Lyapunov-Krasovskii functional, delay decomposition method, time-variant delay

## Abstract

Results on finite-gain $\mathcal{L_{\infty}}$ stability from a disturbance to the output of a time-variant delay system are presented via a delay decomposition approach. By constructing an appropriate Lyapunov-Krasovskii functional and a novel integral inequality, which gives a tighter upper bound than Jensen’s inequality and Bessel-Legendre inequality, some sufficient conditions are established and desired feedback controllers are designed in terms of the solution to certain LMIs. Compared with the existing results, the obtained criteria are more effective due to the tuning scalars and free-weighting matrices. Numerical examples and their simulations are given to demonstrate the effectiveness of the proposed method.

## Introduction

In the past few decades, a thorough understanding of dynamic systems from an input-output point of view has been an area of ongoing and intensive research [1–10]. The strength of input-output stability theory is that it provides a method for anticipating the qualitative behavior of a feedback system with only rough information as regards the feedback components [1]. Disturbance phenomenon is considered as a kind of exogenous inputs and is frequently a source of generation of oscillation and instability and poor performance and commonly exists in various mechanical, biological, physical, chemical engineering, economic systems. In this setting several natural questions rise: Does the bounded disturbance produce the bounded response (output)? What are the effects on the output of the same system when tuning the parameters? Do the systems have the property of robustness for the disturbance? Basing on studies of input-output stability, we investigate disturbance-output properties, which demonstrate how the disturbance affects the bounded behaviors of system.

The input-output property is mostly discussed by transfer function [9, 10]. To the best of our knowledge, there exists some limitation as regards the method of transfer function to study input-output stability to certain extent. For example, as is mentioned in [9] of page 4, the system with transfer function ${G_{k}}(s) = \frac{1}{{{(s + 1)}^{k}}(s + 1 + s{e^{ - s}})}$ is bounded-input-bounded-output stable for $k \ge4$, even though ${G_{k}}$ has a sequence of poles asymptotic to the imaginary axis. To determine whether one has stability for smaller values of *k* seems to be beyond our present techniques, and therefore it is interesting and challenging to extend Lyapunov stability tools for the analysis of input/disturbance-output stability.

However, there are very little works about the analysis of disturbance-output stability of systems with time-variant delays by constructed Lyapunov functionals. This motivates the present study. Our performance objective is to design feedback gain matrices to guarantee the output of a class of delay system will remain bounded for any bounded disturbance by the Lyapunov-Krasovskii functional method. We will utilize a delay decomposition approach to take information of delayed plant states into full consideration. The bounds of the output vary with the adjustment of parameters. It is also helpful for estimating the upper bound of some cross terms more precisely.

Another feature of our work is the choice of integral inequalities. As is well known, many researchers have devoted much attention to obtaining much tighter bounds of various functions, especially integral terms of quadratic functions to reduce the conservatism in the fields of controlling and engineering. The common mathematical tools are integral inequality and free-weighting matrix method. The most recent researches are based on the Jensen inequality as one of the essential techniques in dealing with the time delay systems to estimate upper bound of time derivative of constructed Lyapunov functional. Currently, there are a few works to analyze the conservatism of Jensen’s gap [11] in order to reduce Jensen’s gap in the use of the Wirtinger inequality [12–14]. Furthermore, a novel integral inequality called the Bessel-Legendre (B-L) inequality has been developed in [15], which encompasses the Jensen inequality and the Wirtinger-based integral inequality. However, the inequalities in [14] and [15] only concern the study of single integral terms of quadratic functions, while the upper bounds of double integral terms should also be estimated if triple integral terms are introduced in the Lyapunov-Krasovskii functional to reduce the conservatism. It is worth noting that the B-L inequality has only been applied to a stability analysis of the system with constant delay.

In this paper, a new class of integral inequalities for quadratic functions in [16] via intermediate terms called auxiliary functions are introduced to develop the criteria of finite-gain $\mathcal{L_{\infty}}$ stability from a disturbance to the output for systems with time-variant delay and constant delay using appropriate Lyapunov-Krasovskii functionals. These inequalities can produce much tighter bounds than what the above inequalities produce. Moreover, by introducing free-weighting matrix and tuning parameters, feedback gain matrices are obtained. Finally, two numerical examples show efficacy of the proposed approach. Specially, the terms on the left side of the equation

$$\begin{aligned} 2\eta\bigl({x^{T}}(t) + {\dot{x}^{T}}(t)\bigr)N\bigl((A + C{K_{1}})x(t) + (B + C{K_{2}})x\bigl(t - h(t)\bigr) + Cw(t) - \dot{x}(t)\bigr) = 0 \end{aligned}$$

are added to the derivative of the Lyapunov-Krasovskii functional, $V(t)$. In this equation, the free-weighting matrix *N* and the scalar *η* indicate the relationship between the terms in our system and guarantee the negative definite of stability criteria. As is shown in our theorem, they can be determined easily by solving the corresponding linear matrix inequalities.

### Notations

Throughout this paper, ${A^{ - 1}}$ and ${A^{T}}$ stand for the inverse and transpose of a matrix *A*, respectively; $P>0$ ($P\geq0$, $P<0$, $P\leq0$) means that the matrix *P* is symmetric positive definite (positive-semi definite, negative definite and negative-semi definite); $R^{n}$ denotes *n*-dimensional Euclidean space; $R^{{m}\times{n}}$ is the set of $m\times n$ real matrices; $\Vert x \Vert $, $\Vert A \Vert $ denote the Euclidean norm of the vector *x* and the induced matrix norm of *A*, respectively; $\lambda_{\max}(Q)$ and $\lambda_{\min }(Q)$ denote, respectively, the maximal and minimal eigenvalue of a symmetric matrix *Q*.

## Problem statement and preliminaries

Consider the control system with time delay

1 $$ \textstyle\begin{cases} \dot{x}(t) = Ax(t) + Bx(t - h(t)) + C(u(t)+ w(t)),\\ y(t)= Dx(t),\\ x(t) = \phi(t),\quad - {h_{2}} \leq t \leq 0, \end{cases} $$

where $x(t), u(t), y(t), w(t)\in{R^{n}}$ are the state vector, control input, control output, disturbance of the system, respectively; $\phi(t):[ - {h_{2}},0] \to {R^{n}}$ is a continuously differentiable function, $A,B,C,D \in{R^{n \times n}}$ are known real parameter matrices, and $h(t):R \to R$ is a continuous function satisfying

$$\begin{gathered} 0 \leq{h_{1}} \leq h(t) \leq{h_{2}}, \end{gathered} $$

where ${h_{1}}$, ${h_{2}}$ are constants.

Let ${h_{12}} = {h_{2}} - {h_{1}}$, and ${\Vert \phi \Vert _{ - {h_{2}}}}$, ${\Vert {\dot{\phi}} \Vert _{ - {h_{2}}}}$ be defined by ${\Vert \phi \Vert _{ - {h_{2}}}} = \sup _{ - {h_{2}} \leq \theta \leq 0} \Vert {\phi(\theta)} \Vert $, ${\Vert {\dot{\phi}} \Vert _{ - {h_{2}}}} = \sup_{ - {h_{2}} \leq \theta \leq 0} \Vert {\dot{\phi}(\theta)} \Vert $. To obtain the bounded output of system (1), we let

2 $$\begin{aligned} u(t) = {K_{1}}x(t) + {K_{2}}x\bigl(t - h(t)\bigr), \end{aligned}$$

where ${K_{1}}$, ${K_{2}}$ are the feedback gain matrices. Substituting (2) into (1) gives

3 $$ \textstyle\begin{cases} \dot{x}(t)= (A + C{K_{1}})x(t) + (B + C{K_{2}})x(t - h(t)) + Cw(t),\\ y(t)= Dx(t),\\ x(t) = \phi(t),\quad - {h_{2}} \leq t \leq 0. \end{cases} $$

Let us introduce the following definitions and lemmas for later use.

### Definition 2.1

We have a real-valued vector $w(t) \in\mathcal{L}_{\infty}^{n} $, if $\Vert w \Vert _{{\mathcal{L}_{\infty}}} = \sup_{t_{0} \le t < \infty} \Vert {w(t)} \Vert < + \infty$.

### Definition 2.2

The control system (3) is said to be finite-gain $\mathcal{L_{\infty}}$ stable from a disturbance (here *w*) to the output (here *y*) if there exist nonnegative constants *γ* and *θ* such that

$${\bigl\Vert {y(t)} \bigr\Vert } \le\gamma{ \Vert w \Vert _{{\mathcal{L}_{\infty}}}} + \theta $$

for all $w(t) \in\mathcal{L}_{\infty}^{n}$, $t \ge{t_{0}}$.

### Remark 2.1

Definition 2.2 relates the output of the system directly to the disturbance; namely, if the system is finite-gain $\mathcal{L_{\infty}}$ stable from *w* to *y*, then, for every bounded disturbance $w(t)$, the output $y(t)$ is bounded. There is defined according to Definition 5.1 [17] a concept of stability in the input-output sense. The constant *θ* in Definition 2.2 is called the bias term.

### Remark 2.2

The norm function captures the ‘size’ of the signals. The ∞-norm is useful when amplitude constraints are imposed on a problem, and the 2-norm is of more help in the context of energy constraints. We will typically be interested in measuring signals of the ∞-norm.

### Lemma 2.1

[16]

*For a positive definite matrix* $R > 0$, *and a differentiable function* $x(u)$, $u \in[a,b]$, *the following inequalities hold*:

4 $$\begin{aligned} &{\int_{a}^{b} {{{\dot{x}}^{T}}(\alpha)R\dot{x}(\alpha)\,d\alpha} \geq\frac{1}{{b - a}}\Omega_{5}^{T}R{ \Omega_{5}} + \frac{3}{{b - a}}\Omega_{6}^{T}R{ \Omega_{6}} ,} \end{aligned}$$

5 $$\begin{aligned} &{\int_{a}^{b} {{{\dot{x}}^{T}}(\alpha)R\dot{x}(\alpha)\,d\alpha} \geq\frac{1}{{b - a}}\Omega_{5}^{T}R{ \Omega_{5}} + \frac{3}{{b - a}}\Omega_{6}^{T}R{ \Omega_{6}} + \frac{5}{{b - a}}\Omega_{7}^{T}R{ \Omega_{7}} ,} \end{aligned}$$

6 $$\begin{aligned} &{\int_{a}^{b} { \int_{\beta}^{b} {{{\dot{x}}^{T}}(\alpha)R \dot{x}(\alpha)\,d\alpha} \geq2\Omega_{8}^{T}R{\Omega _{8}} + 4\Omega_{9}^{T}R{\Omega _{9}}} ,} \end{aligned}$$

7 $$\begin{aligned} &{\int_{a}^{b} { \int_{a}^{\beta}{{{\dot{x}}^{T}}(\alpha)R \dot{x}(\alpha)\,d\alpha} \geq2\Omega_{10}^{T}R{\Omega _{10}} + 4\Omega_{11}^{T}R{\Omega _{11}}},} \end{aligned}$$

*where*

$$\begin{aligned} &{\Omega_{5}} = x(b) - x(a), \\ &{\Omega_{6}} = x(b) + x(a) - \frac{2}{{b - a}} \int_{a}^{b} {x(\alpha)\,d\alpha} , \\ &{\Omega_{7}} = x(b) - x(a) + \frac{6}{{b - a}} \int_{a}^{b} {x(\alpha)\,d\alpha} - \frac{{12}}{{{{(b - a)}^{2}}}} \int_{a}^{b} { \int_{\beta}^{b} {x(\alpha)} } \,d\alpha\,d\beta, \\ &{\Omega_{8}} = x(b) - \frac{1}{{b - a}} \int_{a}^{b} {x(\alpha)\,d\alpha} , \\ &{\Omega_{9}} = x(b) + \frac{2}{{b - a}} \int_{a}^{b} {x(\alpha)\,d\alpha} - \frac{6}{{{{(b - a)}^{2}}}} \int_{a}^{b} { \int_{\beta}^{b} {x(\alpha)} } \,d\alpha\,d\beta, \\ &{\Omega_{10}} = x(a) - \frac{1}{{b - a}} \int_{a}^{b} {x(\alpha)\,d\alpha} , \\ &{\Omega_{11}} = x(a) - \frac{4}{{b - a}} \int_{a}^{b} {x(\alpha)\,d\alpha} + \frac{6}{{{{(b - a)}^{2}}}} \int_{a}^{b} { \int_{\beta}^{b} {x(\alpha)} } \,d\alpha\,d\beta. \end{aligned}$$

### Remark 2.3

Inequalities (4)-(7) can produce much tighter bounds than what the mentioned inequalities produce. Inequality (5) is will be used frequently in the proof of the theorem and the corollary.

### Lemma 2.2

[18] Reciprocal convexity lemma

*For any vector* ${x_{1}}$, ${x_{2}}$, *matrices* $R > 0,S$, *and real scalars* $\alpha \geq 0$, $\beta \geq 0$ *satisfying* $\alpha + \beta = 1$, *the following inequality holds*:

$$-\frac{1}{\alpha }x_{1}^{T}Rx_{1}-\frac{1}{\beta }x_{2}^{T}Rx_{2}\leq -{\left[\begin{array}{c} x_{1}\\ x_{2} \end{array}\right]}^{T}\left[\begin{array}{cc} R & S\\ S^{T} & R \end{array}\right]\left[\begin{array}{c} x_{1}\\ x_{2} \end{array}\right]$$

*subject to*

$$0<\left[\begin{array}{cc} R & S\\ S^{T} & R \end{array}\right].$$

## Main results

In this section, basing on the delay decomposition approach and integral inequality (5), we will give a less conservative criterion such that the time-variant delay system (3) is finite-gain $\mathcal{L_{\infty}}$ stable from *w* to *y*. We will solve the design problem for the feedback controller via LMIs.

### Theorem 3.1

*Given scalars* $0 \leq {h_{1}} \leq {h_{2}}$, *the control system* (3) *with feedback gain matrix* ${K_{1}}$, ${K_{2}}$ *is finite*-*gain* $\mathcal{L_{\infty}}$ *stable from* *w* *to* *y*, *if there exist matrices* $0 < P$, $0 < {Q_{i}}$, $0 < {R_{i}}$, $i = 1, \ldots ,4$, *and* *N*, $S_{ij}$, $i,j = 1, \ldots,4$, *scalars* $0 \le{\varepsilon _{1}}$, $0 \le{\varepsilon_{2}}$, $0 < {\alpha_{1}} < 1$, $0 < {\alpha_{2}} < 1$ *and* *η* *such that*

8 $$\begin{aligned} {\Xi_{(17n \times17n)}} < 0, \end{aligned}$$

*where*

$$\begin{aligned} &{{\Xi_{11}} = {({\alpha_{1}} {h_{1}})^{2}} {R_{1}} + {\bigl((1 - {\alpha_{1}}){h_{1}} \bigr)^{2}} {R_{2}} + {\bigl((1 - {\alpha_{2}}){h_{12}} \bigr)^{2}} {R_{3}} + {({\alpha_{2}} {h_{12}})^{2}} {R_{4}} - \eta N - \eta {N^{T}} + {\varepsilon_{1}} {\eta^{2}}I,} \\ &{{\Xi_{12}} = P - \eta{N^{T}} + \eta NA + \eta {X_{1}},\qquad {\Xi_{17}} = \eta NB + \eta{X_{2}},} \\ &{{\Xi_{22}} = {Q_{1}} + \eta{A^{T}} {N^{T}} + \eta NA + \eta{X_{1}} + \eta X_{1}^{T} + {\varepsilon_{2}} {\eta^{2}}I,} \\ &{{\Xi_{23}} = 3{R_{1}},\qquad {\Xi_{27}} = \eta NB + \eta{X_{2}},\qquad {\Xi_{28}} = - 12{R_{1}},\qquad {\Xi _{29}} = 5{R_{1}},} \\ &{{\Xi_{33}} = - {Q_{1}} + {Q_{2}} - 9{R_{1}} - 9{R_{2}},\qquad {\Xi_{34}} = 3{R_{2}},\qquad {\Xi_{38}} = 18{R_{1}},\qquad {\Xi_{39}} = - 5{R_{1}},} \\ &{{\Xi_{3,10}} = - 12{R_{2}},\qquad {\Xi_{3,11}} = 5{R_{2}},\qquad {\Xi_{44}} = - {Q_{2}} + {Q_{3}} - 9{R_{2}} - 9{R_{4}},\qquad {\Xi_{45}} = 3{R_{4}},} \\ &{{\Xi_{4,10}} = 18{R_{2}},\qquad {\Xi_{4,11}} = - 5{R_{2}},\qquad {\Xi_{4,16}} = - 12{R_{4}},\qquad {\Xi_{4,17}} = 5{R_{4}},} \\ &{{\Xi_{5,5}} = - {Q_{3}} + {Q_{4}} - 9{R_{3}} - 9{R_{4}},\qquad {\Xi_{5,6}} = - S_{21}^{T},\qquad {\Xi _{5,7}} = - S_{11}^{T} + 3{R_{3}},} \\ &{{\Xi_{5,12}} = - S_{31}^{T},\qquad {\Xi_{5,13}} = - S_{41}^{T},\qquad {\Xi_{5,14}} = - 12{R_{3}},\qquad {\Xi_{5,15}} = 5{R_{3}},\qquad {\Xi _{5,16}} = 18{R_{4}},} \\ &{{\Xi_{5,17}} = - 5{R_{4}},\qquad {\Xi_{66}} = - {Q_{4}} - 9{R_{3}},\qquad {\Xi_{67}} = - {S_{22}} + 3{R_{3}},\qquad {\Xi _{6,12}} = 18{R_{3}},} \\ &{{\Xi_{6,13}} = - 5{R_{3}},\qquad {\Xi_{6,14}} = - {S_{23}},\qquad {\Xi _{6,15}} = - {S_{24}},} \\ &{{\Xi_{77}} = - {S_{12}} - S_{12}^{T} - 18{R_{3}},\qquad {\Xi_{7,12}} = - S_{32}^{T} - 12{R_{3}},\qquad {\Xi_{7,13}} = - S_{42}^{T} + 5{R_{3}},} \\ &{{\Xi_{7,14}} = - {S_{13}} + 18{R_{3}},\qquad {\Xi _{7,15}} = - {S_{14}} - 5{R_{3}},\qquad {\Xi _{88}} = - 48{R_{1}},} \\ &{{\Xi_{89}} = 15{R_{1}},\qquad {\Xi_{99}} = - 5{R_{1}},} \\ &{{\Xi_{10,10}} = - 48{R_{2}},\qquad {\Xi_{10,11}} = 15{R_{2}},\qquad {\Xi_{11,11}} = - 5{R_{2}},} \\ &{{\Xi_{12,12}} = - 48{R_{3}},\qquad {\Xi_{12,13}} = 15{R_{3}},\qquad {\Xi_{12,14}} = - {S_{33}},} \\ &{{\Xi_{12,15}} = - {S_{34}},\qquad {\Xi_{13,13}} = - 5{R_{3}},\qquad {\Xi_{13,14}} = - {S_{43}},\qquad {\Xi _{13,15}} = - {S_{44}},} \\ &{{\Xi_{14,14}} = - 48{R_{3}},\qquad {\Xi_{14,15}} = 15{R_{3}},} \\ &{{\Xi_{15,15}} = - 5{R_{3}},\qquad {\Xi_{16,16}} = - 48{R_{4}},\qquad {\Xi_{16,17}} = 15{R_{4}},\qquad {\Xi _{17,17}} = - 5{R_{4}}.} \end{aligned}$$

*The remaining entries are zero and*

9 $$\left[\begin{array}{cccccccc} 9R_{3} & -3R_{3} & 12R_{3} & -5R_{3} & S_{11} & S_{12} & S_{13} & S_{14}\\ -3R_{3} & 9R_{3} & -18R_{3} & 5R_{3} & S_{21} & S_{22} & S_{23} & S_{24}\\ 12R_{3} & -18R_{3} & 48R_{3} & -15R_{3} & S_{31} & S_{32} & S_{33} & S_{34}\\ -5R_{3} & 5R_{3} & -15R_{3} & 5R_{3} & S_{41} & S_{42} & S_{43} & S_{44}\\ S_{11}^{T} & S_{21}^{T} & S_{31}^{T} & S_{41}^{T} & 9R_{3} & -3R_{3} & 12R_{3} & -5R_{3}\\ S_{12}^{T} & S_{22}^{T} & S_{32}^{T} & S_{42}^{T} & -3R_{3} & 9R_{3} & -18R_{3} & 5R_{3}\\ S_{13}^{T} & S_{23}^{T} & S_{33}^{T} & S_{43}^{T} & 12R_{3} & -18R_{3} & 48R_{3} & -15R_{3}\\ S_{14}^{T} & S_{24}^{T} & S_{34}^{T} & S_{44}^{T} & -5R_{3} & 5R_{3} & -15R_{3} & 5R_{3} \end{array}\right]>0.$$

*The desired control gain matrices are given by* ${K_{i}} = {C^{ - 1}}{N^{ - 1}}{X_{i}}$.

### Proof

Consider a Lyapunov-Krasovskii functional candidate

$$\begin{aligned} V(t) = \sum_{i = 1}^{5} {{V_{i}}(t)}, \end{aligned}$$

where

$$\begin{aligned} &{{V_{1}}(t) = {x^{T}}(t)Px(t),} \\ &{{V_{2}}(t) = \int_{t - {\alpha_{1}}{h_{1}}}^{t} {{x^{T}}(\alpha ){Q_{1}}x(\alpha)} \,d\alpha+ \int_{t - {h_{1}}}^{t - {\alpha_{1}}{h_{1}}} {{x^{T}}(\alpha ){Q_{2}}x(\alpha)} \,d\alpha,} \\ &{{V_{3}}(t) = \int_{t - {h_{3}}}^{t - {h_{1}}} {{x^{T}}(\alpha ){Q_{3}}x(\alpha)} \,d\alpha+ \int_{t - {h_{2}}}^{t - {h_{3}}} {{x^{T}}(\alpha ){Q_{4}}x(\alpha)} \,d\alpha,} \\ &{{V_{4}}(t) = {\alpha_{1}} {h_{1}} \int_{ - {\alpha_{1}}{h_{1}}}^{0} { \int_{t + \beta}^{t} {{{\dot{x}}^{T}}(\alpha ){R_{1}}\dot{x}(\alpha)} } \,d\alpha\,d\beta} \\ &{\phantom{{V_{4}}(t) =} {}+ (1 - {\alpha _{1}}){h_{1}} \int_{ - {h_{1}}}^{ - {\alpha_{1}}{h_{1}}} { \int_{t + \beta}^{t} {{{\dot{x}}^{T}}(\alpha ){R_{2}}\dot{x}(\alpha)} } \,d\alpha\,d\beta,} \\ &{{V_{5}}(t) = (1 - {\alpha_{2}}){h_{12}} \int_{ - {h_{2}}}^{ - {h_{3}}} { \int_{t + \beta}^{t} {{{\dot{x}}^{T}}(\alpha ){R_{3}}\dot{x}(\alpha)} } \,d\alpha\,d\beta} \\ &{\phantom{{V_{5}}(t) =} {}+ {\alpha_{2}} {h_{12}} \int_{ - {h_{3}}}^{ - {h_{1}}} { \int_{t + \beta}^{t} {{{\dot{x}}^{T}}(\alpha ){R_{4}}\dot{x}(\alpha)} } \,d\alpha\,d\beta,} \end{aligned}$$

where ${h_{3}} = {h_{1}} + {\alpha_{2}}{h_{12}}$. Then the time derivative of $V(t)$ along the trajectories of equation (3) is

$$\begin{aligned} \dot{V}(t) = \sum_{i = 1}^{5} {{{\dot{V}}_{i}}(t)} , \end{aligned}$$

where

10 $$\begin{aligned} &{{{\dot{V}}_{1}}(t) =2{{\dot{x}}^{T}}(t)Px(t),} \end{aligned}$$

11 $$\begin{aligned} &{{{\dot{V}}_{2}}(t) ={x^{T}}(t){Q_{1}}x(t) - {x^{T}}(t - {\alpha_{1}} {h_{1}}){Q_{1}}x(t - {\alpha_{1}} {h_{1}}) + {x^{T}}(t - {\alpha _{1}} {h_{1}}){Q_{2}}x(t - {\alpha _{1}} {h_{1}})} \\ &{\phantom{{{\dot{V}}_{2}}(t) =} {} - {x^{T}}(t - {h_{1}}){Q_{2}}x(t - {h_{1}}),} \end{aligned}$$

12 $$\begin{aligned} &{{{\dot{V}}_{3}}(t) ={x^{T}}(t - {h_{1}}){Q_{3}}x(t - {h_{1}}) - {x^{T}}(t - {h_{3}}){Q_{3}}x(t - {h_{3}}) + {x^{T}}(t - {h_{3}}){Q_{4}}x(t - {h_{3}})} \\ &{\phantom{{{\dot{V}}_{3}}(t) =} {}- {x^{T}}(t - {h_{2}}){Q_{4}}x(t - {h_{2}}),} \end{aligned}$$

13 $$\begin{aligned} &{{{\dot{V}}_{4}}(t) ={({\alpha_{1}} {h_{1}})^{2}} {{\dot{x}}^{T}}(t){R_{1}}\dot{x}(t) - {\alpha_{1}} {h_{1}} \int_{t - {\alpha_{1}}{h_{1}}}^{t} {{{\dot{x}}^{T}}(\alpha ){R_{1}}\dot{x}(\alpha)} \,d\alpha+ {({h_{1}} - {\alpha _{1}} {h_{1}})^{2}} {{\dot{x}}^{T}}(t){R_{2}} \dot{x}(t)} \\ &{\phantom{{{\dot{V}}_{4}}(t) =} {} - ({h_{1}} - {\alpha_{1}} {h_{1}}) \int_{t - {h_{1}}}^{t - {\alpha_{1}}{h_{1}}} {{{\dot{x}}^{T}}(\alpha ){R_{2}}\dot{x}(\alpha)} \,d\alpha,} \end{aligned}$$

14 $$\begin{aligned} &{{{\dot{V}}_{5}}(t) ={({h_{2}} - {h_{3}})^{2}} {{\dot{x}}^{T}}(t){R_{3}}\dot{x}(t) - ({h_{2}} - {h_{3}}) \int_{t - {h_{2}}}^{t - {h_{3}}} {{{\dot{x}}^{T}}(\alpha ){R_{3}}\dot{x}(\alpha)} \,d\alpha+ {({h_{3}} - {h_{1}})^{2}} {{\dot{x}}^{T}}(t){R_{4}} \dot{x}(t)} \\ &{\phantom{{{\dot{V}}_{5}}(t) =} {}- ({h_{3}} - {h_{1}}) \int_{t - {h_{3}}}^{t - {h_{1}}} {{{\dot{x}}^{T}}(\alpha ){R_{4}}\dot{x}(\alpha)} \,d\alpha.} \end{aligned}$$

Applying the proposed integral inequality (5) in Lemma 2.1 leads to

15 $$\begin{aligned} &{- {\alpha_{1}} {h_{1}} \int_{t - {\alpha_{1}}{h_{1}}}^{t} {{{\dot{x}}^{T}}(\alpha ){R_{1}}\dot{x}(\alpha)} \,d\alpha} \\ &{\quad \leq-{\Gamma^{T}}(t) \bigl\{ {({e_{2}} - {e_{3}}){R_{1}} {({e_{2}} - {e_{3}})^{T}}} + 3({e_{2}} + {e_{3}} - {e_{8}}){R_{1}} {({e_{2}} + {e_{3}} - {e_{8}})^{T}}} \\ &{\qquad{} + 5({e_{2}} - {e_{3}} + 3{e_{8}} - {e_{9}}){R_{1}} {({e_{2}} - {e_{3}} + 3{e_{8}} - {e_{9}})^{T}} \bigr\} \Gamma(t),} \end{aligned}$$

16 $$\begin{aligned} &{- (1 - {\alpha_{1}}){h_{1}} \int_{t - {h_{1}}}^{t - {\alpha_{1}}{h_{1}}} {{{\dot{x}}^{T}}(\alpha ){R_{2}}\dot{x}(\alpha)} \,d\alpha} \\ &{\quad \leq- {\Gamma^{T}}(t) \bigl\{ {({e_{3}} - {e_{4}}){R_{2}} {({e_{3}} - {e_{4}})^{T}}} + 3({e_{3}} + {e_{4}} - {e_{10}}){R_{2}} {({e_{3}} + {e_{4}} - {e_{10}})^{T}}} \\ &{\qquad {} + 5({e_{3}} - {e_{4}} + 3{e_{10}} - {e_{11}}){R_{2}} {({e_{3}} - {e_{4}} + 3{e_{10}} - {e_{11}})^{T}} \bigr\} \Gamma(t),} \end{aligned}$$

17 $$\begin{aligned} &{- {\alpha_{2}} {h_{12}} \int_{t - {h_{3}}}^{t - {h_{1}}} {{{\dot{x}}^{T}}(\alpha ){R_{4}}\dot{x}(\alpha)} \,d\alpha} \\ &{\quad \leq- {\Gamma^{T}}(t) \bigl\{ {({e_{4}}-{e_{5}}){R_{4}} {({e_{4}} - {e_{5}})^{T}}} + 3({e_{4}} + {e_{5}} - {e_{16}}){R_{4}} {({e_{4}} + {e_{5}} - {e_{16}})^{T}} } \\ &{\qquad {} + 5({e_{4}} - {e_{5}} + 3{e_{16}} - {e_{17}}){R_{4}} {({e_{4}} - {e_{5}} + 3{e_{16}} - {e_{17}})^{T}} \bigr\} \Gamma(t),} \end{aligned}$$

where

$$\begin{array}{rl} \Gamma (t)= & [\begin{array}{ccccccc} \dot{x}(t) & x(t) & x(t-\alpha_{1}h_{1}) & x(t-h_{1}) & x(t-h_{3}) & x(t-h_{2}) & x(t-h(t)) \end{array}\\ & \begin{array}{ccc} \frac{2}{\alpha_{1}h_{1}}\int_{t-\alpha_{1}h_{1}}^{t}x(\alpha )\;d\alpha & \frac{12}{{\left(\alpha_{1}h_{1}\right)}^{2}}\int_{t-\alpha_{1}h_{1}}^{t}\int_{\beta }^{t}x(\alpha )\;d\alpha \;d\beta & \frac{2}{h_{1}-\alpha_{1}h_{1}}\int_{t-h_{1}}^{t-\alpha_{1}h_{1}}x(\alpha )\;d\alpha \end{array}\\ & \begin{array}{cc} \frac{12}{{\left(h_{1}-\alpha_{1}h_{1}\right)}^{2}}\int_{t-h_{1}}^{t-\alpha_{1}h_{1}}\int_{\beta }^{t-\alpha_{1}h_{1}}x(\alpha )\;d\alpha \;d\beta & \frac{2}{h_{2}-h(t)}\int_{t-h_{2}}^{t-h(t)}x(\alpha )\;d\alpha \end{array}\\ & \begin{array}{cc} \frac{12}{{\left(h_{2}-h(t)\right)}^{2}}\int_{t-h_{2}}^{t-h(t)}\int_{\beta }^{t-h(t)}x(\alpha )\;d\alpha \;d\beta & \frac{2}{h(t)-h_{3}}\int_{t-h(t)}^{t-h_{3}}x(\alpha )\;d\alpha \end{array}\\ & \begin{array}{cc} \frac{12}{{\left(h(t)-h_{3}\right)}^{2}}\int_{t-h(t)}^{t-h_{3}}\int_{\beta }^{t-h_{3}}x(\alpha )\;d\alpha \;d\beta & \frac{2}{\alpha_{2}h_{12}}\int_{t-h_{3}}^{t-h_{1}}x(\alpha )\;d\alpha \end{array}\\ & \begin{array}{c} \frac{12}{{\left(\alpha_{2}h_{12}\right)}^{2}}\int_{t-h_{3}}^{t-h_{1}}\int_{\beta }^{t-h_{1}}x^{T}(\alpha )\;d\alpha \;d\beta {]}^{T}, \end{array} \end{array}$$

${e_{i}}(i = 1,2, \ldots,17) \in{R^{17n \times n}}$ are elementary matrices, for example

$$e_{1}^{T}=\left[\begin{array}{ccccccccccccccccc} I & 0 & 0 & 0 & 0 & 0 & 0 & 0 & 0 & 0 & 0 & 0 & 0 & 0 & 0 & 0 & 0 \end{array}\right].$$

Furthermore, there are two cases about $h(t)$, ${h_{3}} \leq h(t) \leq {h_{2}}$, or ${h_{1}} \leq h(t) \leq {h_{3}}$. We only discuss the first case, and the other case can be discussed similarly.

*Case* 1: ${h_{3}} \leq h(t) \leq {h_{2}}$.

In fact,

$$\begin{aligned} \int_{t - {h_{2}}}^{t - {h_{3}}} {{{\dot{x}}^{T}}(\alpha ){R_{3}}\dot{x}(\alpha)} \,d\alpha= \int_{t - {h_{2}}}^{t - h(t)} {{{\dot{x}}^{T}}(\alpha ){R_{3}}\dot{x}(\alpha)} \,d\alpha+ \int_{t - h(t)}^{t - {h_{3}}} {{{\dot{x}}^{T}}(\alpha ){R_{3}}\dot{x}(\alpha)} \,d\alpha. \end{aligned}$$

So, by Lemma 2.1 again, we get

18 $$\begin{aligned} &{- (1 - {\alpha_{2}}){h_{12}} \int_{t - {h_{2}}}^{t - h(t)} {{{\dot{x}}^{T}}(\alpha ){R_{3}}\dot{x}(\alpha)} \,d\alpha} \\ &{\quad \leq- \frac{{(1 - {\alpha _{2}}){h_{12}}}}{{{h_{2}} - h(t)}}{\Gamma ^{T}}(t) \bigl\{ {({e_{7}} - {e_{6}}){R_{3}} {({e_{7}} - {e_{6}})^{T}}}} \\ &{\qquad{} {}+ 3({e_{7}} + {e_{6}} - {e_{12}}){R_{3}} {({e_{7}} + {e_{6}} - {e_{12}})^{T}}} \\ &{\qquad{} + 5({e_{7}} - {e_{6}} + 3{e_{12}} - {e_{13}}){R_{3}} {({e_{7}} - {e_{6}} + 3{e_{12}} - {e_{13}})^{T}} \bigr\} \Gamma(t),} \end{aligned}$$

19 $$\begin{aligned} &{- (1 - {\alpha_{2}}){h_{12}} \int_{t - h(t)}^{t - {h_{3}}} {{{\dot{x}}^{T}}(\alpha ){R_{3}}\dot{x}(\alpha)} \,d\alpha} \\ &{\quad \leq- \frac{{(1 - {\alpha _{2}}){h_{12}}}}{{h(t) - {h_{3}}}}{\Gamma ^{T}}(t) \bigl\{ {({e_{5}} - {e_{7}}){R_{3}} {({e_{5}} - {e_{7}})^{T}}}} \\ &{\qquad{} + 3({e_{5}} + {e_{7}} - {e_{14}}){R_{3}} {({e_{5}} + {e_{7}} - {e_{14}})^{T}}} \\ &{\qquad{} + 5({e_{5}} - {e_{7}} + 3{e_{14}} - {e_{15}}){R_{3}} {({e_{5}} - {e_{7}} + 3{e_{14}} - {e_{15}})^{T}} \bigr\} \Gamma(t).} \end{aligned}$$

Using Lemma 2.2, we obtain the following relation from equations (18) and (19):

20 $$\begin{array}{c} -(1-\alpha_{2})h_{12}\int_{t-h_{2}}^{t-h(t)}{\dot{x}}^{T}(\alpha )R_{3}\dot{x}(\alpha )\;d\alpha -(1-\alpha_{2})h_{12}\int_{t-h(t)}^{t-h_{3}}{\dot{x}}^{T}(\alpha )R_{3}\dot{x}(\alpha )\;d\alpha \\ \;\leq -\frac{(1-\alpha_{2})h_{12}}{h_{2}-h(t)}x_{1}^{T}\Omega x_{1}-\frac{(1-\alpha_{2})h_{12}}{h(t)-h_{3}}x_{2}^{T}\Omega x_{2}\\ \;\leq -{\left[\begin{array}{c} x_{1}\\ x_{2} \end{array}\right]}^{T}\left[\begin{array}{cc} \Omega & S\\ S^{T} & \Omega \end{array}\right]\left[\begin{array}{c} x_{1}\\ x_{2} \end{array}\right] \end{array}$$

subject to (9) defined in Theorem 3.1, where

$$\begin{array}{c} x_{1}=\mathrm{col}\left\{\begin{array}{cc} \left[\begin{array}{c} x(t-h(t))\\ x(t-h_{2}) \end{array}\right] & \left[\begin{array}{c} \frac{2}{h_{2}-h(t)}\int_{t-h_{2}}^{t-h(t)}x(\alpha )\;d\alpha \\ \frac{12}{{\left(h_{2}-h(t)\right)}^{2}}\int_{t-h_{2}}^{t-h(t)}\int_{\beta }^{t-h(t)}x(\alpha )\;d\alpha \;d\beta \end{array}\right] \end{array}\right\},\\ x_{2}=\mathrm{col}\left\{\begin{array}{cc} \left[\begin{array}{c} x(t-h_{3})\\ x(t-h(t)) \end{array}\right] & \left[\begin{array}{c} \frac{2}{h(t)-h_{3}}\int_{t-h(t)}^{t-h_{3}}x(\alpha )\;d\alpha \\ \frac{12}{{\left(h(t)-h_{3}\right)}^{2}}\int_{t-h(t)}^{t-h_{3}}\int_{\beta }^{t-h_{3}}x(\alpha )\;d\alpha \;d\beta \end{array}\right] \end{array}\right\},\\ \Omega =\left[\begin{array}{cccc} 9R_{3} & -3R_{3} & 12R_{3} & -5R_{3}\\ -3R_{3} & 9R_{3} & -18R_{3} & 5R_{3}\\ 12R_{3} & -18R_{3} & 48R_{3} & -15R_{3}\\ -5R_{3} & 5R_{3} & -15R_{3} & 5R_{3} \end{array}\right],\;S=\left[\begin{array}{cccc} S_{11} & S_{12} & S_{13} & S_{14}\\ S_{12}^{T} & S_{22} & S_{23} & S_{24}\\ S_{13}^{T} & S_{23}^{T} & S_{33} & S_{34}\\ S_{14}^{T} & S_{24}^{T} & S_{34}^{T} & S_{44} \end{array}\right]. \end{array}$$

Moreover, for any scalars ${\varepsilon_{1}} > 0$, ${\varepsilon_{2}} > 0$, we have

21 $$\begin{aligned} &{2\eta{{\dot{x}}^{T}}(t)NCw(t) \le{\varepsilon_{1}} {\eta ^{2}} {{\dot{x}}^{T}}(t)\dot{x}(t) + {{1 \over {{\varepsilon_{1}}}}} {r^{T}}(t){C^{T}} {N^{T}}NCw(t),} \end{aligned}$$

22 $$\begin{aligned} &{2\eta{x^{T}}(t)NCw(t) \le{\varepsilon_{2}} {\eta ^{2}} {x^{T}}(t)x(t) + {{1 \over {{\varepsilon_{2}}}}} {r^{T}}(t){C^{T}} {N^{T}}NCw(t).} \end{aligned}$$

Combining equations (10)-(22) gives

$$\begin{aligned} \dot{V}(t)&\le{\Gamma^{T}}(t)\Xi\Gamma(t) - 9{x^{T}}(t){R_{1}}x(t) + \biggl({{1 \over {{\varepsilon_{1}}}}} + {{1 \over {{\varepsilon_{2}}}}} \biggr){w^{T}}(t){C^{T}} {N^{T}}NCw(t) \\ &\le- 9{\lambda_{\min}}({R_{1}}){\bigl\Vert {x(t)} \bigr\Vert ^{2}} + \biggl({{1 \over {{\varepsilon_{1}}}}} + { {1 \over {{\varepsilon_{2}}}}}\biggr){\Vert {NC} \Vert ^{2}}\Vert w \Vert ^{2}_{\mathcal{L}_{\infty}}. \end{aligned}$$

Let ${c_{1}} = 9{\lambda_{\min}}({R_{1}})$, ${c_{2}} = ({{1 \over {{\varepsilon_{1}}}}} + {{1 \over {{\varepsilon_{2}}}}}){\Vert {NC} \Vert ^{2}}\Vert w \Vert ^{2}_{\mathcal{L}_{\infty}}$, we have

$$\begin{aligned} \dot{V}(t) \le- {c_{1}} {\bigl\Vert {x(t)} \bigr\Vert ^{2}} + {c_{2}}. \end{aligned}$$

Now we shall show that the state $x(t)$ is bounded for $t \ge0$.

First suppose ${\Vert {x(t)} \Vert ^{2}} \ge{{{{c_{2}}} \over {{c_{1}}}}}$ for $t \ge0$. Then $V(t) \le V(0)$ for all $t \ge0$, which implies

$${\bigl\Vert {x(t)} \bigr\Vert ^{2}} \le{{{V(t)} \over {{\lambda _{\min}}(P)}}} \le{{{V(0)} \over {{\lambda_{\min}}(P)}}} \le{{{{d_{1}}\Vert \phi \Vert _{ - {h_{2}}}^{2} + {d_{2}}\Vert {\dot{\phi}} \Vert _{ - {h_{2}}}^{2}} \over {{\lambda_{\min}}(P)}}}, $$

where

$$\begin{aligned} &{{d_{1}}={\lambda_{\max}}(P) + {\alpha_{1}} {h_{1}} {\lambda_{\max}}({Q_{1}}) + (1 - {\alpha _{1}}){h_{1}} {\lambda_{\max}}({Q_{2}}) + {\alpha_{2}} {h_{12}} {\lambda_{\max}}({Q_{3}})} \\ &{\phantom{{d_{1}}=} {}+ (1 - {\alpha_{2}}){h_{12}} {\lambda_{\max}}({Q_{4}}),} \\ &{{d_{2}}={{1 \over 2}} {({\alpha_{1}} {h_{1}})^{3}} {\lambda_{\max}}({R_{1}}) + {{1 \over 2}}(1 + {\alpha_{1}}){(1 - {\alpha _{1}})^{2}}h_{1}^{3}{\lambda _{\max}}({R_{2}}) } \\ &{\phantom{{d_{2}}=} {}+ {{1 \over 2}}({h_{2}} + {h_{3}}){(1 - {\alpha_{2}})^{2}}h_{12}^{2}{ \lambda_{\max}}({R_{3}})} \\ &{\phantom{{d_{2}}=} {}+ {{1 \over 2}}({h_{1}} + {h_{3}}){({\alpha _{2}} {h_{12}})^{2}} {\lambda_{\max}}({R_{4}}).} \end{aligned}$$

Now consider the case ${\Vert {x(t)} \Vert ^{2}} \le{{{{c_{2}}} \over {{c_{1}}}}}$ for $t \ge0$. Then $x(t)$ is bounded obviously.

If the first two cases were not true, there would exist ${t_{2}} > {t_{1}} > 0$, such that

$${\bigl\Vert {x({t_{1}})} \bigr\Vert ^{2}} < { {{{c_{2}}} \over {{c_{1}}}}},\qquad {\bigl\Vert {x({t_{2}})} \bigr\Vert ^{2}} > {{{{c_{2}}} \over {{c_{1}}}}}, $$

which implies there exists a ${t^{*} } > 0$ due to the continuity of $x(t)$ such that $V({t^{*} }) = \sum_{i = 1}^{5} {{V_{i}}({t^{*} })} $ and $V(t) \le V({t^{*} })$ for $t \in[{t^{*} },{t_{2}}]$.

Thus for $t \in[{t^{*} },{t_{2}}]$, we have

$${\bigl\Vert {x(t)} \bigr\Vert ^{2}} \le{{{V({t^{*} })} \over {{\lambda_{\min}}(P)}}} \le{{{{d_{1}}{{{{c_{2}}} \over {{c_{1}}}}} + {d_{2}}d_{3}^{2}} \over {{\lambda _{\min}}(P)}}}, $$

where

$$\begin{aligned} &{{d_{3}} = \biggl( { \bigl( {\bigl\Vert {(A + C{K_{1}})} \bigr\Vert + \bigl\Vert {(B + C{K_{2}})} \bigr\Vert } \bigr)\sqrt {\biggl({{1 \over {{\varepsilon_{1}}{c_{1}}}}} + {{1 \over {{\varepsilon _{2}}{c_{1}}}}}\biggr){{\Vert {NC} \Vert }^{2}}} + \Vert C \Vert } \biggr){\Vert w \Vert _{{\mathcal{L}_{\infty}}} }} \\ &{\phantom{{d_{3}}} = {e_{1}} {\Vert w \Vert _{{\mathcal {L}_{\infty}}}},} \\ &{{e_{1}} = \bigl( {\bigl\Vert {(A + C{K_{1}})} \bigr\Vert + \bigl\Vert {(B + C{K_{2}})} \bigr\Vert } \bigr)\sqrt{ \biggl({{1 \over {{\varepsilon_{1}}{c_{1}}}}} + {{1 \over {{\varepsilon _{2}}{c_{1}}}}}\biggr){{\Vert {NC} \Vert }^{2}}} + \Vert C \Vert .} \end{aligned}$$

Therefore in the last case, ${\Vert {x(t)} \Vert ^{2}} \le\max \{ {\frac{{{c_{2}}}}{{{c_{1}}}},{{{{d_{1}}{{{{c_{2}}} \over {{c_{1}}}}} + {d_{2}}d_{3}^{2}} \over {{\lambda_{\min}}(P)}}}} \}$, $t \ge0$.

Note that, for $t \ge0$,

$${\bigl\Vert {x(t)} \bigr\Vert ^{2}} \le{{{{d_{1}}\Vert \phi \Vert _{ - {h_{2}}}^{2} + {d_{2}}\Vert {\dot{\phi}} \Vert _{ - {h_{2}}}^{2}} \over {{\lambda_{\min}}(P)}}} + {{{{c_{2}}} \over {{c_{1}}}}} + {{{{d_{1}}{{{{c_{2}}} \over {{c_{1}}}}} + {d_{2}}d_{3}^{2}} \over {{\lambda_{\min}}(P)}}}. $$

Thus,

$$\begin{aligned} \bigl\Vert {x(t)} \bigr\Vert \le&\sqrt{{{{{d_{1}}\Vert \phi \Vert _{ - {h_{2}}}^{2} + {d_{2}}\Vert {\dot{\phi}} \Vert _{ - {h_{2}}}^{2}} \over {{\lambda_{\min}}(P)}}} + {{{{c_{2}}} \over {{c_{1}}}}} + {{{{d_{1}}{{{{c_{2}}} \over {{c_{1}}}}} + {d_{2}}d_{3}^{2}} \over {{\lambda_{\min}}(P)}}}} \\ \le& \biggl(\biggl({c_{1}}{d_{1}}\Vert \phi \Vert _{ - {h_{2}}}^{2} + {c_{1}}{d_{2}}\Vert {\dot{\phi}} \Vert _{ - {h_{2}}}^{2} + \biggl( \biggl({{1 \over {{\varepsilon_{1}}}}} + {{1 \over {{\varepsilon_{2}}}}}\biggr){{\Vert {NC} \Vert }^{2}}{\lambda_{\min}}(P) \\ &{}+ {d_{1}}\biggl({{1 \over {{\varepsilon _{1}}}}} + {{1 \over {{\varepsilon_{2}}}}}\biggr){{\Vert {NC} \Vert }^{2}} + {c_{1}}{d_{1}}e_{1}^{2} \biggr)\Vert w \Vert ^{2}_{\mathcal{L}_{\infty}}\biggr)\big/\bigl({c_{1}}{\lambda_{\min}}(P)\bigr)\biggr)^{1/2} \\ \le&\frac{{\sqrt{{c_{1}}{d_{1}}} {{\Vert \phi \Vert }_{ - {h_{2}}}} + \sqrt{{c_{1}}{d_{2}}} {{\Vert {\dot{\phi}} \Vert }_{ - {h_{2}}}}}}{{\sqrt{{c_{1}}{\lambda_{\min}}(P)} }} \\ &{}+ \sqrt{\frac{{({{1 \over {{\varepsilon_{1}}}}} + {{1 \over {{\varepsilon_{2}}}}}){{\Vert {NC} \Vert }^{2}}{\lambda_{\min}}(P) + {d_{1}}({{1 \over {{\varepsilon_{1}}}}} + {{1 \over {{\varepsilon _{2}}}}}){{\Vert {NC} \Vert }^{2}} + {c_{1}}{d_{1}}e_{1}^{2}}}{{{c_{1}}{\lambda _{\min}}(P)}}} {\Vert w \Vert _{{\mathcal{L}_{\infty}}} }. \end{aligned}$$

So

$$\begin{aligned} &{\Vert y \Vert \le \Vert D \Vert \bigl\Vert {x(t)} \bigr\Vert } \\ &{\quad \le \frac{{\Vert D \Vert ( {\sqrt{{c_{1}}{d_{1}}} {{\Vert \phi \Vert }_{ - {h_{2}}}} + \sqrt{{c_{1}}{d_{2}}} {{\Vert {\dot{\phi}} \Vert }_{ - {h_{2}}}}} )}}{{\sqrt{{c_{1}}{\lambda_{\min}}(P)} }}} \\ &{\qquad{} + \sqrt{\frac{{({{1 \over {{\varepsilon_{1}}}}} + {{1 \over {{\varepsilon_{2}}}}}){{\Vert {NC} \Vert }^{2}}{\lambda_{\min}}(P) + {d_{1}}({{1 \over {{\varepsilon_{1}}}}} + {{1 \over {{\varepsilon _{2}}}}}){{\Vert {NC} \Vert }^{2}} + {c_{1}}{d_{1}}e_{1}^{2}}}{{{c_{1}}{\lambda _{\min}}(P)}}} \Vert D \Vert {\Vert w \Vert _{{\mathcal{L}_{\infty}}} }.} \end{aligned}$$

Let

$$\begin{aligned} &{{\gamma} = \sqrt{\frac{{({{1 \over {{\varepsilon_{1}}}}} + {{1 \over {{\varepsilon_{2}}}}}){{\Vert {NC} \Vert }^{2}}{\lambda_{\min}}(P) + {d_{1}}({{1 \over {{\varepsilon_{1}}}}} + {{1 \over {{\varepsilon _{2}}}}}){{\Vert {NC} \Vert }^{2}} + {c_{1}}{d_{1}}e_{1}^{2}}}{{{c_{1}}{\lambda _{\min}}(P)}}} \Vert D \Vert ,} \\ &{{\theta} = \frac{{\Vert D \Vert ( {\sqrt{{c_{1}}{d_{1}}} {{\Vert \phi \Vert }_{ - {h_{2}}}} + \sqrt{{c_{1}}{d_{2}}} {{\Vert {\dot{\phi}} \Vert }_{ - {h_{2}}}}} )}}{{\sqrt{{c_{1}}{\lambda_{\min }}(P)} }}.} \end{aligned}$$

This shows the trivial solution of system (3) is finite-gain $\mathcal{L_{\infty}}$ stable from *w* to *y* and the feedback gain matrices ${K_{i}}$, $i = 1,2$ are expressed in the form of ${K_{i}} = {C^{ - 1}}{N^{ - 1}}{X_{i}}$. □

### Remark 3.1

Instead of constructing the state feedback by the pre-determined method, Theorem 3.1 fixes them by solving LMIs. So, suitable ones are always chosen due to the free-weighting *N*, thus overcoming the conservatism of Theorem 3.1 in [4, 5].

### Remark 3.2

The proposed integral inequalities in Lemma 2.1 give much tighter upper bounds in equations (15)-(19) than those obtained by Jensen’s inequality. Therefore, the resulting stability criterion in Theorem 3.1 is much less conservative than the ones based on Jensen’s inequality.

### Remark 3.3

The utilized state-augmented vector $\Gamma(t)$ includes newly proposed double integral terms such as $(12/({\alpha_{1}}{h_{1}})^{2}) \int_{t - {\alpha_{1}}{h_{1}}}^{t} {\int_{\beta}^{t} {{x^{T}}(\alpha)} } \,d\alpha\, d\beta$, $(12/ ( h(t) - {h_{3}} )^{2}) \int_{t - h(t)}^{t - {h_{3}}} \int_{\beta}^{t - {h_{3}}} {x(\alpha)} \,d\alpha\, d\beta$. If $h(t) = h$, the system under the assumption

$$u(t) = {K_{1}}x(t) + {K_{2}}x(t - h) $$

is represented by

23 $$ \textstyle\begin{cases} \dot{x}(t) = Ax(t) + Bx(t - h) + C(u(t)+w(t)),\\ y(t)= Dx(t),\\ x(t) = \phi(t),\quad - h \leq t \leq 0, \end{cases} $$

where ${\Vert \phi \Vert _{ - h}}$, ${\Vert {\dot{\phi}} \Vert _{ - h}}$ are defined by ${\Vert \phi \Vert _{ - h}} = \sup_{ - h \leq \theta \leq 0} \Vert {\phi(\theta)} \Vert $, ${\Vert {\dot{\phi}} \Vert _{ - h}} = \sup_{ - h \leq \theta \leq 0} \Vert {\dot{\phi}(\theta)} \Vert $. Through a similar line as in the proof of Theorem 3.1, we have the following corollary.

### Corollary 3.1

*The control system* (23) *with feedback gain matrix* ${K_{1}}$, ${K_{2}}$ *is finite*-*gain* $\mathcal{L_{\infty}}$ *stable from* *w* *to* *y*, *if there exist matrices* $0 < P$, $0 < {Q_{i}}$, $0 < {R_{i}}$, $i = 1,2$, *and* *N*, *scalars* *η*, $0 \leq {\beta_{1}}$, $0 \leq {\beta_{2}}$, *such that*

24 $$\psi_{\left(10n\times 10n\right)}=\left(\begin{array}{cc} \tilde{\psi } & \varpi \\ \varpi^{T} & \Lambda \end{array}\right)<0,$$

*where*

$$\begin{array}{rl} & \tilde{\psi }\in R_{8n\times 8n},\\ & \varpi_{\left(2n\times 8n\right)}^{T}=\left[\begin{array}{cccccccc} \frac{\eta }{\sqrt{\beta_{1}}}C^{T}N^{T} & 0 & 0 & 0 & 0 & 0 & 0 & 0\\ 0 & \frac{\eta }{\sqrt{\beta_{2}}}C^{T}N^{T} & 0 & 0 & 0 & 0 & 0 & 0 \end{array}\right],\\ & \Lambda_{2n\times 2n}=\left[\begin{array}{cc} -I & 0\\ 0 & -I \end{array}\right],\\ & \psi_{11}={\left(\alpha_{3}h\right)}^{2}R_{1}+{\left((1-\alpha_{3})h\right)}^{2}R_{2}-\eta N-\eta N^{T},\;\psi_{12}=P-\eta N^{T}+\eta NA+\eta X_{1},\\ & \psi_{16}=\eta NB+\eta X_{2},\;\psi_{19}=\frac{\eta }{\sqrt{\beta_{2}}}NC,\;\psi_{22}=Q_{1}+\eta A^{T}N^{T}+\eta NA+\eta X_{1}+\eta X_{1}^{T},\\ & \psi_{23}=3R_{1},\;\psi_{24}=-12R_{1},\;\psi_{25}=5R_{1},\;\psi_{26}=\eta NB+\eta X_{2},\\ & \psi_{2,10}=\frac{\eta }{\sqrt{\beta_{1}}}NC,\;\psi_{33}=-Q_{1}+Q_{2}-9R_{1}-9R_{2},\;\psi_{34}=18R_{1},\;\psi_{35}=-5R_{1},\\ & \psi_{36}=3R_{2},\;\psi_{37}=-12R_{2},\;\psi_{38}=5R_{2},\;\psi_{44}=-48R_{1},\;\psi_{45}=15R_{1},\\ & \psi_{5,5}=-5R_{1},\;\psi_{66}=-Q_{2}-9R_{2},\;\psi_{67}=18R_{2},\;\psi_{68}=-5R_{2},\\ & \psi_{77}=-48R_{2},\;\psi_{78}=15R_{2},\;\psi_{88}=-5R_{2},\;\psi_{99}=-I,\;\psi_{10,10}=-I. \end{array}$$

*The remaining entries are zero*. *The desired control gain matrices are given by* ${K_{i}} = {C^{ - 1}}{N^{ - 1}}{X_{i}}$.

### Proof

Consider the following Lyapunov-Krasovskii functional candidate:

$$\begin{aligned} V(t) = \sum_{i = 1}^{5} {{V_{i}}(t)}, \end{aligned}$$

where

$$\begin{aligned} &{V_{1}}(t) = {x^{T}}(t)Px(t), \\ &{V_{2}}(t) = \int_{t - {\alpha_{3}}h}^{t} {{x^{T}}(\alpha ){Q_{1}}x(\alpha)} \,d\alpha, \\ &{V_{3}}(t) = \int_{t - h}^{t - {\alpha_{3}}h} {{x^{T}}(\alpha ){Q_{2}}x(\alpha)} \,d\alpha, \\ &{V_{4}}(t) = {\alpha_{3}}h \int_{ - {\alpha_{3}}h}^{0} { \int_{t + \beta}^{t} {{{\dot{x}}^{T}}(\alpha ){R_{1}}\dot{x}(\alpha)} } \,d\alpha\,d\beta, \\ &{V_{5}}(t) = (1 - {\alpha_{3}})h \int_{ - h}^{ - {\alpha_{3}}h} { \int_{t + \beta}^{t} {{{\dot{x}}^{T}}(\alpha ){R_{2}}\dot{x}(\alpha)} } \,d\alpha\,d\beta. \end{aligned}$$

The time derivative along the trajectories of equations (23) is

25 $$\begin{aligned} \dot{V}(t) = &2{{\dot{x}}^{T}}(t)Px(t) + {x^{T}}(t){Q_{1}}x(t) - {x^{T}}(t - {\alpha_{3}}h){Q_{1}}x(t - {\alpha _{3}}h) \\ &{}+ {x^{T}}(t - {\alpha_{3}}h){Q_{2}}x(t - {\alpha _{3}}h) - {x^{T}}(t - h){Q_{2}}x(t - h) \\ &{}+ {({\alpha_{3}}h)^{2}} {{\dot{x}}^{T}}(t){R_{1}} \dot{x}(t) - {\alpha_{3}}h \int_{t - {\alpha_{3}}h}^{t} {{{\dot{x}}^{T}}(\alpha ){R_{1}}\dot{x}(\alpha)} \,d\alpha \\ &{}+ {\bigl((1 - {\alpha_{3}})h\bigr)^{2}} {{\dot{x}}^{T}}(t){R_{2}}\dot{x}(t) - (1 - {\alpha_{3}})h \int_{t - h}^{t - {\alpha_{3}}{h_{1}}} {{{\dot{x}}^{T}}(\alpha ){R_{2}}\dot{x}(\alpha)} \,d\alpha \\ &{}+ {\bigl(2\eta x(t) + 2\eta\dot{x}(t)\bigr)^{T}}N\bigl((A + C{K_{1}})x(t) + (B + C{K_{2}})x(t - h) + Cw(t) - \dot{x}(t) \bigr). \end{aligned}$$

By inequality (5) in Lemma 2.1, we obtain

26 $$\begin{aligned} &{-{\alpha_{3}}h \int_{t - {\alpha_{3}}h}^{t} {{{\dot{x}}^{T}}(\alpha ){R_{1}}\dot{x}(\alpha)} \,d\alpha} \\ &{\quad \leq- {\gamma^{T}}(t) \bigl\{ {({{\overset{\hbox{$\smash{\scriptscriptstyle\frown }$}}{e} }_{2}} - {{\overset{\hbox{$\smash{\scriptscriptstyle \frown}$}}{e} }_{3}}){R_{1}} {({{\overset{ \hbox{$\smash{\scriptscriptstyle\frown}$}}{e} }_{2}} - {{\overset{ \hbox{$\smash{\scriptscriptstyle\frown}$}}{e} }_{3}})^{T}}}} \\ &{\qquad{} + 3({{\overset{\hbox{$\smash{\scriptscriptstyle\frown}$}}{e} }_{2}} + {{\overset{\hbox{$\smash{\scriptscriptstyle \frown}$}}{e} }_{3}} - {{\overset{\hbox{$\smash{ \scriptscriptstyle\frown}$}}{e} }_{4}}){R_{1}} {({{ \overset{\hbox{$\smash{\scriptscriptstyle\frown}$}}{e} }_{2}} + {{\overset{\hbox{$\smash{\scriptscriptstyle \frown}$}}{e} }_{3}} - {{\overset{\hbox{$\smash{ \scriptscriptstyle\frown}$}}{e} }_{4}})^{T}}} \\ &{\qquad{} + 5({{\overset{\hbox{$\smash{\scriptscriptstyle\frown}$}}{e} }_{2}} - {{\overset{\hbox{$\smash{\scriptscriptstyle \frown}$}}{e} }_{3}} + 3{{\overset{\hbox{$\smash{ \scriptscriptstyle\frown}$}}{e} }_{4}} - {{\overset{\hbox{$ \smash{\scriptscriptstyle\frown}$}}{e} }_{5}}){R_{1}} {({{ \overset{\hbox{$\smash{\scriptscriptstyle\frown}$}}{e} }_{2}} - {{\overset{\hbox{$\smash{\scriptscriptstyle \frown}$}}{e} }_{3}} + 3{{\overset{\hbox{$\smash{ \scriptscriptstyle\frown}$}}{e} }_{4}} - {{\overset{\hbox{$ \smash{\scriptscriptstyle\frown}$}}{e} }_{5}})^{T}} \bigr\} \gamma(t),} \end{aligned}$$

27 $$\begin{aligned} &{- (1 - {\alpha_{3}})h \int_{t - h}^{t - {\alpha_{3}}h} {{{\dot{x}}^{T}}(\alpha ){R_{2}}\dot{x}(\alpha)} \,d\alpha} \\ &{\quad \leq- {\gamma^{T}}(t) \bigl\{ {({{\overset{\hbox{$\smash{\scriptscriptstyle\frown }$}}{e} }_{3}} - {{\overset{\hbox{$\smash{\scriptscriptstyle \frown}$}}{e} }_{6}}){R_{2}} {({{\overset{ \hbox{$\smash{\scriptscriptstyle\frown}$}}{e} }_{3}} - {{\overset{ \hbox{$\smash{\scriptscriptstyle\frown}$}}{e} }_{6}})^{T}}}} \\ &{\qquad{} +3({{\overset{\hbox{$\smash{\scriptscriptstyle\frown}$}}{e} }_{3}} + {{\overset{\hbox{$\smash{\scriptscriptstyle \frown}$}}{e} }_{6}} - {{\overset{\hbox{$\smash{ \scriptscriptstyle\frown}$}}{e} }_{7}}){R_{2}} {({{\overset{ \hbox{$\smash{\scriptscriptstyle\frown}$}}{e} }_{3}} + {{ \overset{\hbox{$\smash{\scriptscriptstyle\frown}$}}{e} }_{6}} - {{\overset{\hbox{$\smash{\scriptscriptstyle \frown}$}}{e} }_{7}})^{T}}} \\ &{\qquad{} + 5({{\overset{\hbox{$\smash{\scriptscriptstyle\frown}$}}{e} }_{3}} - {{\overset{\hbox{$\smash{\scriptscriptstyle \frown}$}}{e} }_{6}} + 3{{\overset{\hbox{$\smash{ \scriptscriptstyle\frown}$}}{e} }_{7}} - {{\overset{\hbox{$ \smash{\scriptscriptstyle\frown}$}}{e} }_{8}}){R_{2}} {({{ \overset{\hbox{$\smash{\scriptscriptstyle\frown}$}}{e} }_{3}} - {{\overset{\hbox{$\smash{\scriptscriptstyle \frown}$}}{e} }_{6}} + 3{{\overset{\hbox{$\smash{ \scriptscriptstyle\frown}$}}{e} }_{7}} - {{\overset{\hbox{$ \smash{\scriptscriptstyle\frown}$}}{e} }_{8}})^{T}} \bigr\} \gamma(t),} \end{aligned}$$

where

$$\begin{array}{c} \gamma (t)=\mathrm{col}\left\{\begin{array}{cc} \left[\begin{array}{c} \dot{x}(t)\\ x(t)\\ x(t-\alpha_{3}h)\\ \frac{2}{\alpha_{3}h}\int_{t-\alpha_{3}h}^{t}x(\alpha )\;d\alpha \end{array}\right] & \left[\begin{array}{c} \frac{12}{{\left(\alpha_{3}h\right)}^{2}}\int_{t-\alpha_{3}h}^{t}\int_{\beta }^{t}x(\alpha )\;d\alpha \;d\beta \\ x(t-h)\\ \frac{2}{(1-\alpha_{3})h}\int_{t-h}^{t-\alpha_{3}h}x(\alpha )\;d\alpha \\ \frac{12}{{\left((1-\alpha_{3})h\right)}^{2}}\int_{t-h}^{t-\alpha_{3}h}\int_{\beta }^{t-\alpha_{3}h}x(\alpha )\;d\alpha \;d\beta \end{array}\right] \end{array}\right\}, \end{array}$$

$$\begin{array}{c} {\overset{⌢}{e}}_{i}(i=1,2,\ldots ,8)\in R^{8n\times n}\;\text{are elementary matrices, for example}\\ {\overset{⌢}{e}}_{1}^{T}=[\begin{array}{cccccccc} I & 0 & 0 & 0 & 0 & 0 & 0 & 0 \end{array}]. \end{array}$$

For ${\beta_{1}} \in R\backslash\{ 0\}$, ${\beta_{2}} \in R\backslash\{ 0\} $, it is clear that

28 $$\begin{aligned} &{2\eta{{\dot{x}}^{T}}(t)NCw(t) - {\beta_{1}} {w^{T}}(t)w(t) + {\beta_{1}} {w^{T}}(t)w(t)} \\ &{\quad =- {\beta_{1}} { \biggl[ {w(t) - \frac{\eta}{{{\beta_{1}}}}{C^{T}} {N^{T}}\dot{x}(t)} \biggr]^{T}} \biggl[ {w(t) - \frac{\eta}{{{\beta_{1}}}}{C^{T}} {N^{T}}\dot{x}(t)} \biggr]} \\ &{\qquad{} + \frac{{{\eta^{2}}}}{{{\beta_{1}}}}{{\dot{x}}^{T}}(t)NC{C^{T}} {N^{T}} \dot{x}(t) + {\beta_{1}} {w^{T}}(t)w(t),} \end{aligned}$$

29 $$\begin{aligned} &{2\eta{x^{T}}(t)NCw(t) - {\beta_{2}} {w^{T}}(t)w(t) + {\beta_{2}} {w^{T}}(t)w(t)} \\ &{\quad =- {\beta_{2}} { \biggl[ {w(t) - \frac{\eta}{{{\beta_{2}}}}{C^{T}} {N^{T}}x(t)} \biggr]^{T}} \biggl[ {w(t) - \frac{\eta}{{{\beta_{2}}}}{C^{T}} {N^{T}}\dot{x}(t)} \biggr]} \\ &{\qquad{} + \frac{{{\eta^{2}}}}{{{\beta_{2}}}}{x^{T}}(t)NC{C^{T}} {N^{T}}x(t) + {\beta_{2}} {w^{T}}(t)w(t).} \end{aligned}$$

This, together with (25)-(27), shows we have

$$\begin{aligned} \dot{V}(t) =&2{{\dot{x}}^{T}}(t)Px(t) + {x^{T}}(t){Q_{1}}x(t) - {x^{T}}(t - {\alpha_{3}}h){Q_{1}}x(t - {\alpha _{3}}h) \\ &{}+ {x^{T}}(t - {\alpha_{3}}h){Q_{2}}x(t - {\alpha_{3}}h) \\ &{}-{x^{T}}(t - h){Q_{2}}x(t - h)+ {({\alpha _{3}}h)^{2}} {{\dot{x}}^{T}}(t){R_{1}} \dot{x}(t) - {\alpha_{3}}h \int_{t - {\alpha_{3}}h}^{t} {{{\dot{x}}^{T}}(\alpha ){R_{1}}\dot{x}(\alpha)} \,d\alpha \\ &{}+{\bigl(2\eta x(t) + 2\eta\dot{x}(t)\bigr)^{T}}N\bigl((A + C{K_{1}})x(t) + (B + C{K_{2}})x(t - h) + Cw(t) - \dot{x}(t) \bigr) \\ =& {\gamma^{T}}(t) \bigl(\tilde{\psi}- \varpi{\Lambda ^{ - 1}} {\varpi^{T}}\bigr)\gamma(t) - 9{x^{T}}(t){R_{1}}w(t) + {\beta_{1}} {w^{T}}(t)w(t) + {\beta_{2}} {w^{T}}(t)w(t) \\ \le&- 9{x^{T}}(t){R_{1}}w(t) + {\beta_{1}} {w^{T}}(t)w(t) + {\beta_{2}} {w^{T}}(t)w(t) \\ \le&- 9{\lambda_{\min}}({R_{1}}){\bigl\Vert {x(t)} \bigr\Vert ^{2}} + ({\beta_{1}} + {\beta_{2}}) \Vert w \Vert ^{2}_{\mathcal{L}_{\infty}} \end{aligned}$$

if condition (24) holds. Thus the output of systems (23) can be expressed as

$${\bigl\Vert {y(t)} \bigr\Vert } \le{{\tilde{\gamma}}} {\Vert w \Vert _{{\mathcal{L}_{\infty}}}} + {{\tilde{\theta}}}, $$

where

$$\begin{aligned} &{{{\tilde{\gamma}}} = \sqrt{\frac{{({\beta_{1}} + {\beta_{2}}){\lambda _{\min}}(P) + {{\tilde{d}}_{1}}({\beta_{1}} + {\beta_{2}}) + {c_{1}}{{\tilde{d}}_{2}}\tilde{e}_{2}^{2}}}{{{c_{1}}{\lambda_{\min}}(P)}}} \Vert D \Vert ,} \\ &{\tilde{\theta}= \frac{{\Vert D \Vert ( {\sqrt{{c_{1}}{{\tilde{d}}_{1}}} {{\Vert \phi \Vert }_{ - h}} + \sqrt{{c_{1}}{{\tilde{d}}_{2}}} {{\Vert {\dot{\phi}} \Vert }_{ - h}}} )}}{{\sqrt {{c_{1}}{\lambda_{\min}}(P)} }},} \\ &{{c_{1}} = 9{\lambda_{\min}}({R_{1}}),\qquad {{\tilde{d}}_{1}} = {\lambda_{\max}}(P) + {\alpha_{3}}h{ \lambda_{\max}}({Q_{1}}) + (1 - {\alpha_{3}})h{ \lambda_{\max}}({Q_{2}}),} \\ &{{{\tilde{d}}_{2}} = {{1 \over 2}} {({\alpha _{3}}h)^{3}} {\lambda_{\max}}({R_{1}}) + {{1 \over 2}}(1 + {\alpha_{3}}) \bigl(1 - {\alpha _{3}}^{2}\bigr){h^{2}} {\lambda _{\max}}({R_{2}}),} \\ &{{{\tilde{d}}_{3}} = {{\tilde{e}}_{2}} {\Vert w \Vert _{\mathcal{L}_{\infty}}},\qquad {{\tilde{e}}_{2}} = \bigl( {\bigl\Vert {(A + C{K_{1}})} \bigr\Vert + \bigl\Vert {(B + C{K_{2}})} \bigr\Vert } \bigr)\sqrt{{{{({\beta_{1}} + {\beta_{2}})} \over {{c_{1}}}}}} + \Vert C \Vert .} \end{aligned}$$

The feedback gain matrices ${K_{i}}$, $i = 1,2$, are expressed as ${K_{i}} = {C^{ - 1}}{N^{ - 1}}{X_{i}}$. □

### Remark 3.4

To reduce the conservatism, equivalent transformations are employed through the positive scalars ${\beta _{1}}$, ${\beta_{2}}$ and the free-weighting matrix *N* instead of using inequalities when dealing with the item of ${x^{T}}(t)NCw(t)$ and ${\dot{x}^{T}}(t)NCw(t)$ in Corollary 3.1. As is shown in (28) and (29), the terms ${\dot{x}^{T}}(t)NCw(t)$ and ${{{{\eta^{2}}} \over {{\beta_{2}}}}}{\dot{x}^{T}}(t)NC{C^{T}}{N^{T}}\dot{x}(t)$ and all the resulting relations in $\dot{V}(t) = \sum_{i = 1}^{5} {{{\dot{V}}_{i}}(t)} $ are well used and stability criteria are given in the form of LMIs.

### Remark 3.5

The bound of output $y(t)$ is dependent on feedback gain matrices in Theorem 3.1 and Corollary 3.1. That is to say, the bound of output $y(t)$ can be adjusted by our free weighing matrix *N*. In this way, our results are much less conservative than those in [4, 5]. To this end, the control problem has been solved in terms of a solution to the LMIs (8) and (24).

### Remark 3.6

By developing a delay decomposition approach, the information of delayed plant states can be taken into full consideration. It is worth pointing out that this method has been more widely adopted to the discussion of neural networks and less conservatism is realized by choosing different Lyapunov matrices in the decomposed integral intervals and estimating the upper bound of some cross terms more exactly. It is easily extended to disturbance-output properties of linear time-varying delay systems and the bound of output is influenced by tuning parameters, which will be illustrated with two numerical examples. Since the delay term is concerned more exactly, less conservative results are presented.

## Examples

In this section, two numerical examples are provided to show the effectiveness of the proposed method.

### Example 4.1

As an application of Theorem 3.1, we consider the system (3) with the following parameters:

$$A=\left[\begin{array}{cc} 0.0 & 1.0\\ -100 & -1.0 \end{array}\right],\;B=\left[\begin{array}{cc} 0 & 0.1\\ 0.1 & -1 \end{array}\right],\;C=\left[\begin{array}{cc} 2 & 1\\ 1 & 0 \end{array}\right],\;D=\left[\begin{array}{cc} 1 & 3\\ 0 & 2 \end{array}\right].$$

As the Remark 3.5 states, the bound of output $y(t)$ is dependent on the feedback gain matrices which are solutions to certain LMIs related to parameters *η*, ${\varepsilon_{1}}$, ${\varepsilon_{2}}$, ${\alpha _{1}}$, ${\alpha_{2}}$. For $h(t) = 1.10365 + 1.00995\sin t$, ${w^{T}}(t) = [\sin t\ \cos t]$, $\eta = 0.0508$, ${\varepsilon_{1}} = 0.4849$, ${\varepsilon_{2}} = 0.3935$, the stabilizing control gain matrices ${K_{1}}$, ${K_{2}}$ can easily be solved by LMI (8) and (9) with ${\alpha_{1}} = 0.6551$, ${\alpha_{2}} = 0.1626$. We have

$$K_{1}=\left[\begin{array}{cc} 90.2095 & -35.3177\\ -183.8310 & 70.7326 \end{array}\right],\;K_{2}=\left[\begin{array}{cc} -0.1001 & 1.0002\\ 0.1995 & -2.0997 \end{array}\right].$$

**Figure 1:**
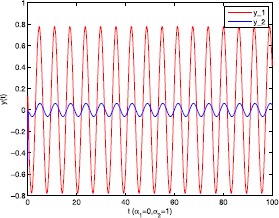
The output in Example 4.1 with $\alpha _{1} = 0$, $\alpha _{2} = 1$.

**Figure 2:**
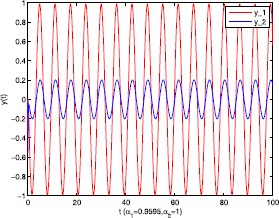
The output in Example 4.1 with $\alpha _{1} = 0.9595$, $\alpha _{2} = 1$.

**Figure 3:**
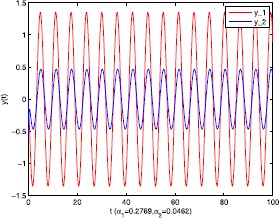
The output in Example 4.1 with $\alpha _{1} = 0.2769$, $\alpha _{2} = 0.0462$.

**Figure 4:**
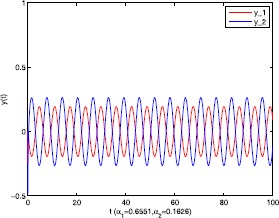
The output in Example 4.1 with $\alpha _{1} = 0.6551$, $\alpha _{2} = 0.1626$.

Figures 1 to 4 show that we can use the method of delay decomposition to vary the bound of output. Figure 1 shows the bound of output without any delay decomposition, while Figure 2 shows the larger bound of output with one delay decomposition, that is, ${\alpha_{1}} = 0.9595$. We also can get a much larger bound of output given in Figure 3 by ${\alpha_{1}} = 0.2769$, ${\alpha_{2}} = 0.0462$ and a much smaller one given in Figure 4 by ${\alpha_{1}} = 0.6551$, ${\alpha_{2}} = 0.1626$.

### Example 4.2

Consider the system (23) with

$$A=\left[\begin{array}{cc} -4 & 1\\ 1 & -4 \end{array}\right],\;B=\left[\begin{array}{cc} -2 & 0\\ 0 & -1 \end{array}\right],\;C=\left[\begin{array}{cc} 2 & 1\\ 1 & 0 \end{array}\right],\;D=\left[\begin{array}{cc} 1 & 3\\ 0 & 2 \end{array}\right].$$

The purpose is to show the bound of output can be adjusted by delay decomposition and to compare the allowable bounds of time delay *h* that guarantee the boundedness of the above system. For $w^{T}(t)=[\begin{array}{cc} \sin(t) & \cos(t) \end{array}]$, solving LMI (24) gives us the stabilizing feedback gain matrices with $h = 2.555$, ${\alpha_{3}} = 0.7952$, $\eta = - 0.1225$, ${\beta _{1}} = 0.3816$, ${\beta_{2}} = 0.7655$. We have

$$K_{1}=\left[\begin{array}{cc} -9.0100 & -20.5402\\ -0.5402 & -3.9296 \end{array}\right],\;K_{2}=\left[\begin{array}{cc} -0.0041 & 0.9509\\ 1.9509 & -1.9060 \end{array}\right].$$

**Figure 5:**
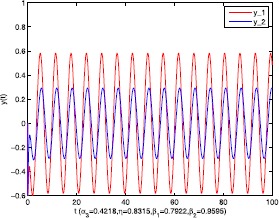
The output in Example 4.2 with $\alpha _{3} = 0.4218$, $\eta = 0.8315$, $\beta _{1} = 0.7922$, $\beta _{2} = 0.9595$.

**Figure 6:**
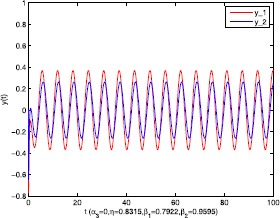
The output in Example 4.2 with $\alpha _{3} = 0$, $\eta = 0.8315$, $\beta _{1} = 0.7922$, $\beta _{2} = 0.9595$.

**Figure 7:**
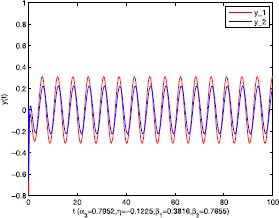
The output in Example 4.2 with $\alpha _{3} = 0.7952$, $\eta = - 0.1225$, $\beta _{1} = 0.3816$, $\beta _{2} = 0.7655$.

**Figure 8:**
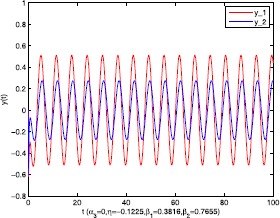
The output in Example 4.2 with $\alpha _{3} = 0$, $\eta = - 0.1225$, $\beta _{1} = 0.3816$, $\beta _{2} = 0.7655$.

The larger bound of output is shown in Figure 5 by ${\alpha_{3}} = 0.4218$ as compared with that without delay decomposition in Figure 6. Certainly, the bound of output can also become smaller shown in Figures 7 and 8. And it can be seen that the proposed approaches can provide a higher bound than that in the existing result [5] with the same parameters.

## Conclusions

In this paper, we consider the disturbance-output property of a delay system. Our contributions are as follows: (1) The delay decomposition approach is used to take information of delayed plant states into full consideration. It is also helpful for estimating the upper bound of some cross terms more precisely. Our examples reveal that we can use this method to vary the bounds of the output by tuning the parameters. (2) Compared with the existing results on the analysis of the input-output stability, our criteria are established by the method of Lyapunov and LMI tools instead of small gain theory or transfer function. We show how Lyapunov stability tools can be used to establish $\mathcal{L}_{\infty}$ stability of dynamic systems represented by the state model. (3) A novel integral inequality is utilized, which produces much tighter bounds than what the Jensen inequality and B-L inequality produce. Potential applications of the theoretical results proposed here need to be developed. Moreover, it is interesting to consider the disturbance-output property by impulsive control in future work.
